# From Therapeutic Drug to Xenobiotic in Cancer Repurposing: Clozapine Mechanisms, Metabolic Liabilities, and Human-Relevant Translational Approaches

**DOI:** 10.3390/jox16040125

**Published:** 2026-07-02

**Authors:** Maria João Gouveia, Nuno Vale

**Affiliations:** 1 PerMed Research Group, RISE-Health, Department of Community Medicine, Health Information and Decision (MEDCIDS), Faculty of Medicine, University of Porto, Alameda Professor Hernâni Monteiro, 4200-319 Porto, Portugal; mariajoaogouveia@gmail.com; 2 Laboratory of Personalized Medicine, Department of Community Medicine, Health Information and Decision (MEDCIDS), Faculty of Medicine, University of Porto, Rua Doutor Plácido da Costa, 4200-450 Porto, Portugal; 3 Department of Community Medicine, Information and Health Decision Sciences (MEDCIDS), Faculty of Medicine, University of Porto, Rua Doutor Placido da Costa, 4200-450 Porto, Portugal

**Keywords:** clozapine, xenobiotics, drug repurposing, oncology, pharmacokinetics, pharmacodynamics, toxicology, drug metabolism, precision oncology, digital twins

## Abstract

Drug repurposing represents a rational and resource-efficient strategy to expand the oncological armamentarium by leveraging the established pharmacology, clinical experience, and safety-monitoring frameworks of approved non-oncological agents. Clozapine (CZP), an atypical antipsychotic characterized by broad receptor pharmacology, complex biotransformation, and clinically relevant toxicological liabilities, has emerged as a candidate of interest following preclinical evidence of context-dependent anticancer activity across multiple tumor types. As such, CZP provides an informative case study at the interface between therapeutic drug action and xenobiotic behavior. This review provides a critical and integrated synthesis of the current evidence supporting the repurposing of CZP in oncology, with particular emphasis on the relationship between its molecular mechanisms, dose–exposure requirements, pharmacological complexity, and potential toxicity. Analysis of in vitro and in vivo studies across glioblastoma, non-small cell lung cancer, breast cancer, and melanoma brain metastasis models indicates that CZP can impair tumor cell proliferation and survival through a form of mechanistic plasticity. Rather than acting through a single conserved pathway, CZP appears to disrupt shared upstream processes related to pro-survival signaling, cellular stress tolerance, and metabolic homeostasis, while engaging tumor-specific downstream responses, including autophagic cell death, mitochondria-dependent apoptosis, oxidative stress, and coordinated modulation of survival and angiogenic pathways. Despite this mechanistic rationale, translation remains substantially constrained, most notably by the order of magnitude gap between anticancer-effective concentrations in vitro and clinically achievable plasma exposures, requiring careful distinction between potentially useful anticancer pharmacology and nonspecific xenobiotic-induced cellular stress and clinically unacceptable toxicity. Key limitations include the discrepancy between anticancer-effective concentrations observed in vitro and exposures achievable during standard psychiatric dosing, the limited understanding of how CZP metabolism and metabolite formation may influence efficacy and toxicity, the absence of integrated pharmacokinetic–pharmacodynamic and toxicokinetic modeling, and the lack of dedicated clinical trial evidence. To address these challenges, this review examines complementary translational strategies, including patient-derived organoids, co-culture systems, microphysiological platforms, pharmacokinetic and toxicological modeling, and computational digital twin frameworks. Together, these approaches may support a biologically informed and risk-aware evaluation of CZP, helping to identify responsive tumor contexts, anticipate exposure-related liabilities, and prioritize rational combination strategies. By integrating therapeutic potential with xenobiotic pharmacology and toxicology, this review positions CZP within the evolving landscape of precision oncology and evidence-driven drug repurposing.

## 1. Introduction

Cancer remains one of the leading causes of morbidity and mortality worldwide, despite significant advances in early diagnosis and targeted therapies [1,2]. Tumor heterogeneity, therapy resistance, and treatment-associated toxicity continue to limit long-term clinical efficacy, highlighting the urgent need for novel therapeutic strategies [3]. In this context, drug repurposing—the identification of new anticancer applications for approved non-oncological drugs—has emerged as a cost-effective and time-efficient approach to accelerate the development of cancer therapies [4]. Despite these advantages, drug repurposing in oncology also faces challenges, including suboptimal dosing, context-dependent efficacy, and translational barriers [5,6].

One area that has attracted increasing attention in this regard is the potential anticancer activity of antipsychotic agents. Epidemiological observations suggesting a lower incidence of cancer in patients with schizophrenia [7,8], combined with experimental evidence of cytotoxic effects of several antipsychotics on cancer cells [9,10,11,12], have provided a biological rationale for their investigation as repurposed oncological agents. Several antipsychotics, including thioridazine, penfluridol, chlorpromazine, and haloperidol, among others, have been reported to modulate key processes implicated in cancer progression [13,14,15,16,17,18]. Their extensively characterized pharmacokinetics properties and clinical safety profiles further render them attractive candidates for repositioning in oncology.

Among these agents, clozapine (CZP) remains comparatively underexplored, despite its uniquely broad receptor pharmacology and emerging experimental evidence supporting its biological relevance across diverse cancer models. Unlike other antipsychotics, CZP exhibits a uniquely broad receptor pharmacology—engaging dopaminergic, serotonergic, adrenergic, and muscarinic systems simultaneously—suggesting a capacity to engage multiple tumor-relevant pathways that may prove advantageous in the context of heterogeneous and treatment-resistant malignancies. This review provides an integrated overview of the current evidence supporting the repurposing of CZP for oncological applications (Section 2), the preclinical mechanistic evidence supporting its anticancer activity (Section 3), the current translational and clinical limitations (Section 4), and the experimental strategies that may bridge the gap between preclinical findings and clinical applicability (Section 5). By framing CZP within the broader context of drug repurposing in oncology, this narrative review aims to provide a balanced assessment of its potential and limitations, and to identify directions for future investigation. Studies were identified through focused searches in PubMed/MEDLINE and Scopus, using keywords related to clozapine, cancer, antitumor activity, pharmacokinetics, toxicity, organoids, and digital twins (DT).

## 2. Clozapine: Pharmacological and Pharmacokinetic Features Supporting Drug Repurposing

**Figure 1:**
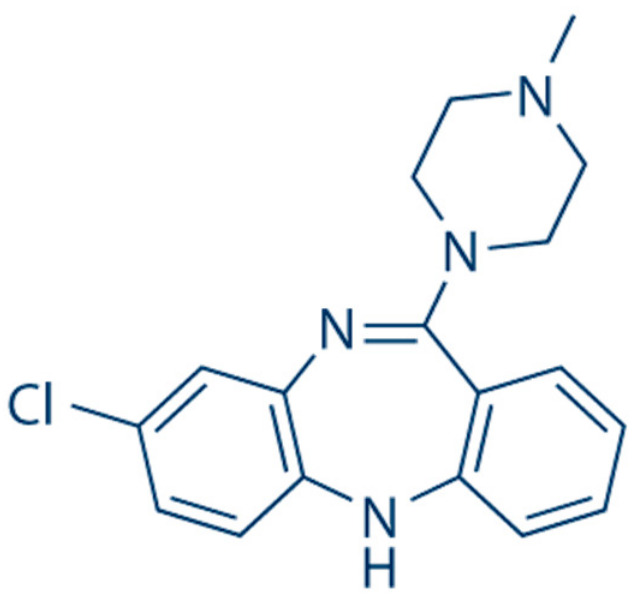
Chemical structure of clozapine, a tricyclic dibenzodiazepine, an FDA-approved antipsychotic in the United States in 1990 [19].

Understanding the pharmacological and pharmacokinetic basis of CZP is essential to evaluate its potential as a repurposed anticancer agent. CZP (Figure 1) is an atypical antipsychotic primarily indicated for treatment-resistant schizophrenia and remains the gold standard for patients unresponsive to other antipsychotic drugs [19,20,21,22]. Unlike typical antipsychotics, whose therapeutic efficacy is largely attributed to dopamine D2 receptor antagonism [23], CZP exhibits a uniquely broad and complex pharmacological profile, engaging with multiple neurotransmitter systems, a feature that extends its biological relevance beyond the central nervous system [19].

CZP displays high binding affinity for several serotonin receptors (5-HT1A, 5-HT2A-C, 5-HT6, 5-HT7), as well as alpha 1 (α1) and alpha 2 (α2) adrenergic receptors, histamine H1 receptors, and muscarinic M1–M5 receptors [24,25,26]. This receptor promiscuity underlies its superior clinical efficacy in psychiatry and its distinct spectrum of biological effects beyond the central nervous system. Importantly, several molecular targets and downstream pathways modulated by CZP intersect with signaling networks critically involved in cancer biology. Dopaminergic and serotonergic pathways have been implicated in the regulation of cell proliferation, apoptosis, angiogenesis, immune modulation, and tumor–stroma interactions [27,28,29,30]. Similarly, α-adrenergic, muscarinic, and histamine receptors are increasingly recognized as contributors to tumor growth, metastatic potential, and therapeutic resistance across multiple cancer types [31,32,33,34,35,36]. Through its multi-receptor engagement, CZP may therefore exert pleiotropic effects on cancer-relevant cellular processes, distinguishing it pharmacologically from more narrowly targeted oncological agents.

From a pharmacokinetic perspective, CZP is characterized by rapid oral absorption, high lipophilicity, extensive tissue distribution, and the ability to cross biological barriers, including the blood–brain barrier [37]. Despite near-complete gastrointestinal absorption, its oral bioavailability is relatively low and highly variable (approximately 27–50%) as a result of pronounced first-pass hepatic metabolism [38]. The drug undergoes extensive hepatic metabolism predominantly via cytochrome P450 enzymes, with CYP1A2 representing the primary metabolic pathway, followed by CYP3A4 and, to a lesser extent, CYP2C19 and CYP2D6. The major circulating metabolite, N-desmethylclozapine (norclozapine), retains pharmacological activity, whereas CZP N-oxide is largely inactive but may undergo reconversion to the parent compound [39,40,41]. The elimination half-life of the drug increases with repeated dosing, reflecting time-dependent pharmacokinetics that may influence systemic exposure during prolonged treatment. CZP exhibits high plasma protein binding (approximately 95–97%) and a large volume of distribution, supporting substantial tissue penetration, a property that may be advantageous in tumor contexts with limited drug accessibility [42,43]. Beyond passive diffusion, CZP’s disposition is also shaped by interactions with ATP-binding cassette (ABC) efflux transporters expressed at the blood–brain barrier (BBB) and other tissue barriers. CZP does not appear to be a strong substrate of P-glycoprotein (P-gp; ABCB1), showing markedly lower in vitro substrate affinity than other atypical antipsychotics such as quetiapine and risperidone [44]; this relatively limited substrate recognition may favor CZP’s efficient penetration across the BBB. CZP does, however, act as an inhibitor of P-gp-mediated efflux in rhodamine-123 transport assays, with potency lower than quetiapine but comparable to other antipsychotics [44,45]. CZP similarly inhibits breast cancer resistance protein (BCRP; ABCG2), a second major efflux transporter present at the BBB and other epithelial barriers, with a half-maximal inhibitory concentration (IC_50_) of approximately 42 µM [46]. Low substrate affinity for both transporters is mechanistically consistent with CZP’s established capacity for efficient CNS tissue penetration.

At the same time, marked interindividual variability in CZP plasma concentrations has been consistently reported and is influenced by multiple factors, including age, sex, smoking status, genetic polymorphisms in CYP enzymes, and ethnic background. In particular, CYP1A2 induction by cigarette smoking can substantially reduce drug exposure, whereas co-administration of CYP1A2 inhibitors (such as fluvoxamine or ciprofloxacin) or CYP3A4 inhibitors can markedly increase plasma levels, heightening the risk of toxicity [47]. These pharmacokinetic characteristics are especially relevant in oncology, where polypharmacy is common and drug–drug interactions with chemotherapeutic agents, targeted therapies, or supportive medications may significantly alter CZP exposure. Collectively, the narrow therapeutic window of CZP, its complex metabolism, and its susceptibility to clinically significant drug–drug interactions underscore the need for careful dose optimization and therapeutic drug monitoring when considering its repositioning for oncological applications. While these properties may pose translational challenges, they also provide a rational framework for patient stratification and exposure-guided dosing strategies in future preclinical and clinical studies. Taken together, CZP’s broad receptor engagement and distinctive pharmacokinetic profile provide a biological and pharmacological rationale for evaluating its repurposing potential. As the following sections describe, this rationale is supported by consistent preclinical signals, although their translational significance remains constrained.

## 3. Preclinical and Mechanistic Evidence of Clozapine’s Anticancer Activity

Building on the pharmacological properties outlined above, a growing body of preclinical research has explored the antitumor potential of CZP across multiple cancer models. Consistent with observations reported for other antipsychotics, emerging evidence suggests that CZP can modulate cellular pathways relevant to cancer biology, with an increasing number of studies describing antitumor activity across diverse experimental systems. Evidence from in vitro systems and selected in vivo studies indicates that CZP consistently impairs tumor cell proliferation and survival in diverse malignancies, although experimental designs, exposure ranges, and tumor contexts vary substantially (Table 1). Translational and clinical evidence, however, remains limited and heterogeneous, a disconnect that highlights the need for the critical and integrated evaluation that follows.

As detailed in Table 1, CZP does not act through a single conserved mechanism; instead, its effects vary according to tumor-specific signaling dependencies and stress-handling capacities. This section integrates these experimental findings with emerging mechanistic insights, providing a unified overview of how CZP perturbs tumor biology in a context-dependent manner.

### 3.1. Central Nervous System Tumor Models: Convergent Disruption of Survival Signaling and Metabolic Homeostasis

Central nervous system models, such as glioblastoma and neuroblastoma models, provided some of the earliest and most mechanistically detailed evidence of CZP antitumor potential [48]. Glioblastoma, characterized by aggressive growth and frequent dysregulation of survival signaling pathways [55], has been a particularly informative model for investigating these effects. In PTEN (Phosphatase and TENsin homolog)-deficient U-87MG human glioblastoma cells, Shin et al. demonstrated that CZP treatment (20–40 µM) significantly suppressed basal and epidermal growth factor (EGF)-induced Akt phosphorylation [48]. This inhibition resulted in dephosphorylation and activation of GSK-3β, decreased cyclin D1 expression, and arrest of cell-cycle progression at G_0_/G_1_ phase, directly linking disruption of survival signaling to impaired proliferative capacity. Notably, these effects were mediated through interference of Ca^2+^/calmodulin-dependent signaling rather than dopamine receptor antagonism, indicating a receptor-independent mechanism that directly interferes with the PI3K/Akt/GSK-3β axis [48]. Notably, CZP’s relatively low affinity as a P-gp substrate compared with other antipsychotics (Section 2) is mechanistically favorable in this context, as P-gp and BCRP are frequently overexpressed in glioblastoma and have been directly implicated in resistance to standard-of-care agents such as temozolomide [56,57]; efflux-mediated resistance is therefore less likely to constrain CZP delivery to tumor cells than it does for many conventional chemotherapeutics. In tumor contexts reliant on constitutive PI3K/Akt activity, such interference appears to constrain proliferative signaling and lower the threshold for subsequent stress responses, rather than directly inducing terminal cell death. Further support for this mechanistic profile emerges from comparative studies examining CZP activity across central nervous system tumor models. In a comparative study using neuroblastoma (SH-SY5Y) and glioblastoma (A172) cell lines, the drug exhibited pronounced cytotoxicity activity, producing significant reductions in cell viability and metabolic activity, with greater sensitivity observed in neuroblastoma cells [49]. Importantly, CZP retained its cytotoxic efficacy under experimentally induced oxidative stress conditions and in co-treatment settings involving modulation of dopamine synthesis through tyrosine supplementation. Analysis of extracellular tyrosine consumption revealed a greater dependence of neuroblastoma cells on tyrosine availability, suggesting differential metabolic dependencies between models and indicating that tumor-specific metabolic context might influence sensitivity to CZP-induced stress [49]. Taken together, findings from glioblastoma and neuroblastoma models demonstrate that CZP consistently impairs proliferative signaling while amplifying cellular stress responses. The convergence of survival pathway disruption and tumor-specific metabolic vulnerability across these models suggests that sensitivity to CZP-induced stress is not incidental but rather reflects an intrinsic dependence on the signaling and metabolic networks that CZP perturbs. Tumors characterized by constitutive PI3K/Akt dependency or heightened metabolic stress may therefore represent particularly vulnerable contexts for CZP-mediated growth suppression.

### 3.2. Melanoma Metastasis Models: Coordinated Disruption of Survival, Inflammatory, and Angiogenic Pathways

Melanoma and melanoma brain metastasis (MBM) models have provided some of the most mechanistically detailed preclinical evidence for CZP antitumor activity within biologically complex and clinically challenging contexts [50]. Given the limited therapeutic options for MBM—a consequence of blood–brain barrier constraints and the distinct immunological and metabolic landscape of the brain [58]—these systems offer a particularly relevant framework to evaluate both anticancer efficacy and tumor selectivity. In human MBM-derived cell lines, CZP inhibited proliferation in a dose-dependent manner (IC_50_ 26–29 µM), while also significantly reducing migratory capacity and clonogenic survival [50]. P-gp and BCRP are known to be expressed in the tumor-associated brain endothelium of MBM, where they have been shown to restrict the brain penetration and efficacy of targeted agents such as BRAF inhibitors [59]; CZP’s comparatively low substrate affinity for these transporters (Section 2) may therefore represent a pharmacokinetic advantage over efflux-susceptible therapeutics in this setting. Non-malignant human astrocytes (NHA) displayed greater tolerance to comparable concentrations, indicating a potential therapeutic window. This difference in tolerance was further supported in three-dimensional fetal rat brain organoids, where toxicity was substantially lower than in MBM cells, suggesting preserved selectivity in a more physiologically relevant microenvironment [50].

Mechanistic studies indicate that CZP perturbs several interconnected pathways that collectively lower the survival capacity of MBM cells. Wikerholmen et al. showed that CZP not only increases apoptosis but also induces a coordinated shift in proteins linked to inflammatory signaling, hypoxia responses, and cell-survival regulation [50]. Upregulation of macrophage inflammatory protein-1α (MIP-1α), interleukin-8 (IL-8), and angiopoietin-like 4 (ANGPTL-4) suggests activation of inflammatory and hypoxia-responsive programs. Conversely, reductions in BCL-x, survivin, and basic fibroblast growth factor point to weakened anti-apoptotic and pro-proliferative support. These tumor alterations—largely absent in non-malignant astrocytes—are consistent with the functional phenotypes already observed, namely reduced proliferation, migration, and clonogenicity together with increased apoptosis [50]. The detection of histamine 4 receptor, muscarinic acetylcholine receptor 3, and nicotinic acetylcholine receptor 5, but not dopamine 4 receptor, further indicates that CZP may act through a combination of receptor-dependent signaling and broader, receptor-independent disruption of stress- and survival-related pathways [50]. These in vitro observations were subsequently extended to an in vivo setting using a metastatic melanoma xenograft (M1/15) model established in athymic nude mice. Systemic administration of CZP (1 mg/kg, subcutaneous) significantly reduced tumor growth and increased median survival compared with vehicle-treated controls [51]. Antitumoral effects were accompanied by a marked reduction in tumor mitotic index, decreased expression of proliferation-associated markers such as proliferating cell nuclear antigen (PCNA), and significant inhibition of intratumoral angiogenesis. Functional expression of the histamine H_4_ receptor in melanoma tissues further linked receptor-level engagement to the observed antitumoral outcomes [51].

Rather than converging on a single tumor-specific molecular target, the evidence from MBM models indicates that CZP induces coordinated modulation of survival, inflammatory, and angiogenic pathways. This pattern of effects appears particularly pronounced within the unique metabolic and immunological context of the brain, reinforcing CZP’s profile as a context-dependent regulator of tumor stress tolerance and growth dynamics. Importantly, these effects were largely absent in non-malignant astrocytes, suggesting that the distinct biological pressures of the brain microenvironment may amplify CZP’s capacity to exploit survival and angiogenic vulnerabilities, identifying MBM as a potentially responsive context for further translational investigation.

### 3.3. Non-Small Cell Lung Cancer: Autophagic Execution of Growth Suppression

The anticancer activity of CZP has also been examined in non-small cell lung cancer (NSCLC), where a distinct cytotoxic profile has been described [52]. In two NSCLC cell lines (A549 and H1299), CZP (0–50 µM) induced a marked, time-dependent inhibition of cell proliferation, accompanied by increased expression of the cyclin-dependent kinase inhibitors p21 and p27, leading to G_0_/G_1_ cell cycle arrest [52]. Notably, these effects occurred in the absence of detectable apoptotic activation, indicating that CZP suppresses NSCLC growth through non-apoptotic mechanisms [52].

Beyond cell-cycle arrest, CZP induced pronounced autophagic activity in both cell lines. Treated A549 and H1299 cells displayed morphological features consistent with autophagy, including intracellular vacuolization and accumulation of autophagosome structures. Biochemically, this was supported by increased LC3-II levels and enhanced autophagic flux, indicating activation of the autophagic machinery rather than mere blockade of autophagosome degradation [52]. The functional significance of this autophagic response was subsequently clarified through pharmacological and genetic approaches. Inhibition of autophagy using bafilomycin A1 and genetic silencing of core autophagy regulators such as Atg7 both partially rescued cell viability [52]. This autophagic execution profile stands in clear contrast to the survival signaling-dependent growth suppression observed in glioblastoma models, suggesting that NSCLC cells harbor a distinct vulnerability to CZP-induced stress that is preferentially channeled through the autophagic machinery. Autophagic tone and flux capacity may therefore represent relevant determinants of responsiveness in this tumor context.

### 3.4. Breast Cancer: Mitochondrial Dysfunction and Intrinsic Apoptotic Vulnerability

Breast cancer models reveal yet another layer of mechanistic diversity in CZP’s anticancer activity, with tumor subtype strongly shaping how cells respond to oxidative stress, mitochondrial dysfunction, and downstream death pathways. In hormone receptor-positive (MCF-7) and triple-negative (MDA-MB-231) breast cancer cell lines, CZP (0–100 µM) reduced cell viability (up to ~60–70% after 72 h), impaired clonogenic survival, and arrested cell-cycle progression at the G_0_/G_1_ phase [51,52]. Across both subtypes, these antiproliferative effects were consistently accompanied by increased intracellular reactive oxygen species (ROS) levels and evidence of mitochondria dysfunction, features that, while shared, are translated into distinct downstream death programs in a subtype-dependent manner.

In MCF-7 cells, CZP inhibited proliferation and clonogenic survival in a dose-dependent manner [53]. This was accompanied by G0/G1 cell-cycle arrest, mediated by downregulation of CDK4, CDK6, and cyclin D1 together with upregulation of the cyclin-dependent kinase inhibitors p21 and p27. ROS emerged as a central upstream mediator linking CZP exposure to apoptotic and autophagic responses: CZP induced a dose-dependent increase in intracellular ROS, accompanied by activation of autophagy (increased LC3-II, Beclin-1, Atg-7, and Atg12/5) and apoptosis. Drug treatment promoted mitochondrial dysfunction together with activation of markers associated with intrinsic apoptosis and autophagy, indicating engagement of multiple stress-responsive pathways [60,61]. Antioxidant pretreatment with α-Tocopherol partially restored CZP-induced cytotoxicity and reduced ROS, autophagy, and apoptosis markers, directly supporting oxidative stress as a key driver of downstream cell death signaling in hormone receptor-positive breast cancer models [54]. Notably, pharmacological inhibition of autophagic flux with chloroquine enhanced rather than reduced CZP-induced cytotoxicity and apoptosis, indicating that autophagy functions primarily as a stress-adaptive survival response in MCF-7 cells rather than as a contributor to cell death, and suggesting that combining CZP with autophagy inhibitors may represent a rational strategy to potentiate its cytotoxic effect in this context [53]. Cytotoxicity in MDA-MB-231 cells was similarly confirmed by Fan et al., who reported ROS- and autophagy-mediated cell death comparable to that observed in MCF-7 cells, with α-Tocopherol pretreatment again rescuing cell viability [53]. Independently, studies evaluating CZP alongside the structurally related antipsychotics cariprazine and olanzapine in the same MDA-MB-231 model provide a complementary, more detailed mechanistic centered on mitochondria-dependent intrinsic apoptosis [54]. CZP exhibited selective cytotoxicity toward MDA-MB-231 cells relative to non-cancerous MRC-5 fibroblasts, with a selectivity index of 3.9. Growth inhibition in this model was associated with a loss of mitochondrial membrane potential and corresponding shift in cytochrome c localization from a predominantly mitochondrial pattern to diffuse cytosol, consistent with activation of the intrinsic apoptotic cascade. These alterations were accompanied by downregulation of anti-apoptotic and pro-survival regulators, including Bcl-2, p62, and phosphorylated Akt, together with upregulation of pro-apoptotic Bax and activation of caspase-3 relative to untreated controls [54]. Notably, in contrast to the autophagy-independent apoptotic profile observed in some other tumor contexts, CZP treatment significantly increased the proportion of autophagic MDA-MB-231 cells (34.7% versus control; *p* = 0.0012), although the accompanying reduction in the autophagic flux marker p62 did not reach statistical significance for CZP specifically, indicating that autophagy is engaged alongside mitochondrial apoptosis but its functional contribution to cell death in this subtype remains less clearly established than in other tumor contexts.

Taken together, these findings indicate that mitochondrial dysfunction represents a shared vulnerability across breast cancer models, while the downstream orchestration of cell death pathways is shaped by subtype-specific stress-handling capacities. In MCF-7 cells, oxidative stress integrates apoptotic and autophagic signaling, whereas in triple-negative models, CZP preferentially engages a mitochondria-dependent apoptotic program with limited involvement of autophagy. This intratumoral heterogeneity further reinforces the context-dependent nature of CZP’s anticancer activity, suggesting that mitochondrial priming status and subtype-specific stress-handling capacities may serve as key determinants of responsiveness. These observations raise important questions about the principles that govern mechanistic diversity across tumor types, questions that are addressed in the following subsection.

### 3.5. Integrative Perspective: Mechanistic Plasticity, Context-Specific Vulnerabilities and Their Translational Significance

**Figure 2:**
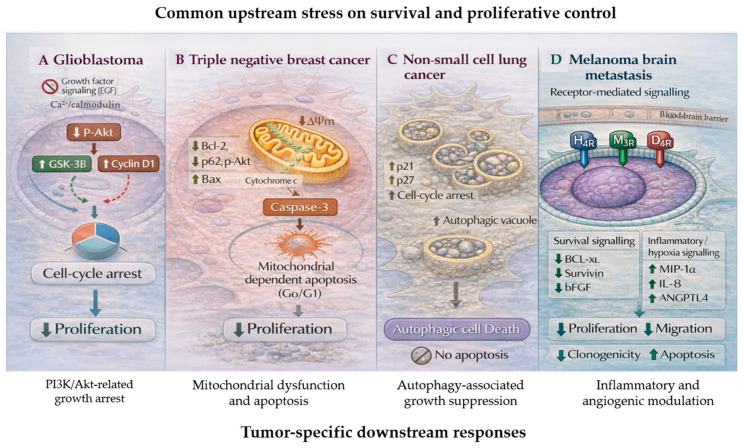
Schematic representation of the context-dependent anticancer activity of CZP across different tumor models. Across the models shown, CZP induces a convergent upstream perturbation of tumor cell fitness, affecting survival signaling, proliferative control, and stress adaptation, while eliciting tumor-specific downstream responses. In glioblastoma (**A**), CZP disrupts PI3K/Akt-dependent survival signaling and cell-cycle regulation, resulting in cell-cycle arrest and reduced proliferation. In triple-negative breast cancer (**B**), mitochondrial integrity is a primary target of CZP, promoting cytochrome c release and activation of intrinsic apoptotic pathways. (**C**) NSCLC cells respond to CZP treatment by activating autophagic cell death mechanisms, leading to suppression of proliferation in the absence of apoptotic activation. In melanoma brain metastasis models (**D**), CZP modulates receptor-mediated signaling involving H_4_R, M_3_R, and D_4_R. This modulation is associated with reduced expression of survival factors (BCL-xL, surviving, and bFGF) and increased levels of inflammatory and hypoxia-associated mediators (MIP-1α, IL-8, and ANGPTL4), ultimately leading to decreased proliferation, migration, and clonogenic potential together with increased apoptotic susceptibility. Together, these findings illustrate the context-dependent nature of CZP anticancer activity and highlight the engagement of distinct stress-response and cell-death programs across tumor types. Figure created with the assistance of generative artificial intelligence (ChatGPT version 5.2, OpenAI).

The preclinical landscape reviewed here raises a central question: how can a single compound suppress tumor growth across such biologically distinct contexts? The answer appears to lie not in a conserved effector mechanism, but in CZP’s capacity to exploit context-specific vulnerabilities within each tumor’s regulatory architecture. Rather than enforcing a uniform mode of cell death, CZP exerts mechanistic plasticity. Specifically, it consistently perturbs upstream processes governing proliferative control, survival signaling, and cellular stress tolerance, while downstream execution pathways diverge markedly across tumor types (Figure 2). This convergence at the level of early growth suppression—frequently manifested as G_0_/G_1_ arrest—suggests that CZP acts primarily as a modulator of stress tolerance and proliferative equilibrium. Beyond this shared upstream disruption, however, downstream responses are highly tumor-specific. While glioblastoma models demonstrate proliferative suppression without consistent commitment to terminal death, NSCLC and breast cancer reveal how the same upstream perturbation can engage fundamentally different executors. Autophagic flux predominates in the former, whereas intrinsic apoptosis—mediated through strong mitochondrial priming—defines the latter. Melanoma brain metastasis models further complicate this picture, as proliferative inhibition in the central nervous system microenvironment is accompanied by coordinated pro-apoptotic and anti-angiogenic effects, likely reflecting the distinct metabolic and immunological pressures of that niche. Ultimately, the nature of the downstream response appears to be shaped by each tumor’s intrinsic signaling dependencies, metabolic state, and stress-handling capacity rather than by a fixed drug-target interaction.

This mechanistic plasticity has important implications for the translational framing of CZP as an anticancer agent. On one hand, the capacity to engage distinct tumor-specific vulnerabilities across diverse cancer types may represent a therapeutic advantage, positioning CZP as a broadly acting stress-modulating agent rather than a narrowly targeted therapy [61,62]. On the other hand, this same heterogeneity implies that predictive biomarkers are unlikely to be universal; their relevance will necessarily be tumor context-dependent, reflecting the distinct molecular and metabolic features that determine responsiveness in each setting [63,64]. Unlike targeted agents, whose efficacy can often be anticipated by the presence of a specific molecular alteration, CZP’s context-dependent activity suggests that responsiveness may be determined by composite features—including mitochondrial priming, autophagic tone, PI3K/Akt dependency, and cell cycle checkpoint integrity—rather than a single actionable target. Defining which tumor contexts are most likely to benefit from CZP-mediated stress modulation, therefore, emerges as a critical prerequisite for rational clinical translation. In this regard, the failure of several repurposed agents in clinical settings has been attributed not to invalid biological hypotheses, but to the evaluation of unselected patient populations in which only molecularly defined subgroups were likely to respond [65,66,67,68]. This underscores the importance of moving beyond broad repurposing strategies toward a more refined framework in which tumor contexts are prospectively stratified according to the features most likely to confer susceptibility to CZP-mediated stress modulation [68].

The convergent mechanistic evidence reviewed above, derived predominantly from in vitro and exploratory in vivo studies, provides a coherent biological basis for continuing to investigate CZP repurposing, rather than firm grounds for anticipating clinical efficacy. The path from mechanistic plausibility to clinical applicability is shaped by important constraints that warrant systematic examination and are addressed in the following section.

## 4. Current Limitations and Translational Challenges

The constraints shaping CZP’s translational trajectory are well-defined and span multiple interconnected domains, encompassing safety and tolerability, pharmacokinetic variability, the absence of pharmacokinetics/pharmacodynamics (PK/PD) modeling linking plasma exposure to antitumor effect, and the lack of direct clinical efficacy data (Table 2). Importantly, these challenges do not stem from a lack of biological activity but rather from issues inherent to CZP’s long-standing psychiatric use, and are therefore well characterized, providing a solid basis for risk-aware translational strategies.

The most prominent safety concern associated with CZP is its risk of hematological toxicity, particularly agranulocytosis and severe neutropenia. This risk was identified early through post-marketing surveillance and led to the implementation of mandatory blood monitoring programs worldwide [69]. Subsequent epidemiological studies and meta-analyses have confirmed that, while the absolute incidence is low, the risk is clinically meaningful and requires continuous vigilance [70]. From a toxicokinetic perspective, the mechanism underlying CZP-induced agranulocytosis is distinct from simple dose-dependent toxicity and reflects the xenobiotic behavior of the drug at the hematopoietic level. CZP undergoes bioactivation by hepatic CYP450 enzymes, myeloperoxidase in peripheral neutrophils, and myeloid precursors in the bone marrow to form a chemically reactive nitrenium ion intermediate [84,85]. This electrophilic species can covalently bind to neutrophil proteins and may exert toxicity through two complementary mechanisms: direct disruption of essential cellular proteins, or haptenization leading to immune-mediated neutrophil destruction [84]. Partial detoxification through glutathione conjugation, yielding C6- and C9-glutathionyl-CZP adducts, may contribute to interindividual variability in susceptibility [85]. Critically, agranulocytosis risk does not appear to correlate clearly with steady-state plasma CZP concentrations [86], which complicates toxicokinetic modeling and suggests that hematological risk is governed more by the local reactive metabolite formation than by systemic exposure alone. This dissociation between plasma levels and hematological toxicity represents a fundamental challenge for any exposure-guided oncological dosing strategy.

CZP is also associated with other adverse effects, including myocarditis, cardiomyopathy, and metabolic disturbances, which are well-documented in psychiatric populations [71,72,73]. The cardiotoxic mechanisms of CZP are multifactorial and likely reflect the convergence of immune-mediated, inflammatory, and direct cellular injury pathways. Proposed mechanisms include IgE-mediated type I hypersensitivity with eosinophilic myocardial infiltration, cytokine release and oxidative stress, catecholaminergic activation resulting from norepinephrine transporter inhibition, and direct activation of cardiomyocyte apoptotic pathways involving mitochondrial dysfunction and NLRP3 inflammasome signaling [87,88]. Notably, recent studies using iPSC-derived cardiomyocytes demonstrated cardiotoxic effects at a physiologically relevant CZP concentration of 2.8 µM, with more pronounced injury in cells derived from patients with a prior history of CZP-induced myocarditis [88]. These findings suggest that susceptibility to cardiotoxicity is not solely concentration-driven but is also shaped by patient-specific biological factors. In an oncological context, these toxicities warrant particular attention, especially in patients receiving myelosuppressive therapies [89]. Notably, most of these risks are dose-dependent, time-dependent, and clinically manageable within established monitoring frameworks.

Beyond safety, CZP displays substantial interindividual pharmacokinetic variability, largely driven by hepatic metabolism via CYP1A2 and influenced by factors such as smoking status, inflammation, and co-medication [74,75]. This variability complicates dose optimization but is not unique to oncology and has been extensively managed in psychiatric practice, offering a transferable foundation for controlled repurposing strategies.

A particularly important translational constraint, insufficiently addressed in the current preclinical literature, is the discrepancy between the concentrations required to elicit anticancer effects in vitro and those achievable during standard psychiatric dosing. As discussed above, anticancer-effective concentrations in vitro typically range from 10 to 100 µM, whereas plasma levels during standard psychiatric treatment generally fall within 0.8–1.8 µM (250–600 ng/mL) [76,77], a discrepancy of one to two orders of magnitude. This gap raises a fundamental question regarding the clinical translatability of the observed in vitro effects, as concentrations substantially exceeding those achievable in vivo may reflect off-target activities that are pharmacologically irrelevant in a clinical context [90]. Consequently, the existing in vitro evidence, while mechanistically informative, cannot be directly extrapolated to clinical settings without further validation at pharmacologically achievable concentrations.

Closely related to the concentration gap is the dose-toxicity issue inherent to any oncological application of CZP. Achieving anticancer-relevant plasma concentrations would likely require doses substantially exceeding those used in psychiatric practice, thereby amplifying the risk of hematological toxicity, cardiomyopathy, and metabolic disturbances in an already vulnerable oncological population [71,72,73]. This creates a narrow—and currently undefined—therapeutic window in which anticancer efficacy might be achievable without unacceptable systemic toxicity. Defining this window represents a critical prerequisite for any rational dose-escalation or repurposing strategy in oncology. Compounding this challenge, conventional preclinical models—including immortalized cell lines and animal systems—are intrinsically limited in their ability to capture human-specific efficacy–toxicity relationships, interindividual pharmacokinetic variability, and patient-specific responses [78,79,80,81], further constraining the translational value of existing preclinical data. To address the concentration-toxicity barrier in CZP repurposing, a translationally relevant strategy is to reduce the dependence on high systemic exposure by combining CZP with existing anticancer agents and by improving tumor selectivity through biomarker-informed and targeted delivery approaches. Combination regimens are particularly important because they may permit antitumor efficacy at lower CZP doses, thereby helping to preserve activity while reducing the risk of dose-limiting hematological and cardiometabolic toxicities [91]. This rationale is supported by preclinical evidence showing that antipsychotic combinations such as chlorpromazine, haloperidol, and risperidone with temozolomide can produce synergistic effects across multiple GBM cell lines [60,92]. As outlined in Section 3, CZP’s anticancer activity is context-dependent and appears to be governed by composite tumor features, including PI3K/Akt dependency, mitochondrial priming status, and autophagic tone [48,49,50,51,52,53,54]. Prospective identification of tumor contexts defined by these signatures, rather than treating all patients as equally likely to respond, could substantially enrich the responsive fraction and reduce the exposure required to achieve a meaningful effect. Accordingly, biomarker-informed patient stratification may further refine the therapeutic index by prospectively identifying the tumor contexts most likely to benefit from such combination regimens [93,94]. Complementarily, targeted delivery systems offer a way to increase intratumoural drug accumulation without proportionally increasing systemic exposure, thereby improving the therapeutic index from a pharmacological standpoint [95,96]. CZP’s high lipophilicity and established compatibility with solid lipid nanoparticle formulations, which have already been characterized in terms of pharmacokinetics, tissue distribution, and bioavailability [43], further support it as a feasible candidate for nanocarrier-based approaches. Together, these approaches provide a mechanistically grounded and clinically plausible framework for overcoming the concentration-toxicity problem and enhancing the translational potential of CZP. A further methodological gap is the absence of integrated pharmacokinetic/pharmacodynamic (PK/PD) modeling linking CZP plasma exposure to antitumor effect. Such models are increasingly recognized as essential tools in oncology drug development, enabling the characterization of exposure–response relationships, the prediction of clinically relevant dose ranges, and the rational design of clinical trials [82]. Their absence in the context of CZP repurposing means that the relationship between achievable plasma concentrations and meaningful antitumor activity remains unquantified, a gap that significantly limits the ability to define a rational therapeutic strategy and to extrapolate preclinical findings to human settings.

Beyond these pharmacological constraints, however, the most consequential gap is the absence of clinical evidence evaluating CZP as an anticancer agent. No clinical trials have been conducted to date, and available human data are limited to retrospective observational studies and pharmacovigilance analyses examining cancer incidence in psychiatric populations treated with CZP, a context fundamentally distinct from its prospective use as an oncological therapy. These studies were not designed to assess anticancer efficacy and are subject to substantial confounding related to underlying disease, lifestyle factors, and treatment duration [83], rendering them insufficient to inform therapeutic conclusions. This gap does not undermine the biological plausibility of CZP anticancer activity but rather defines the central translational challenge that must be addressed before clinical evaluation can be rationally pursued. The following section explores experimental strategies that may contribute to bridging these gaps and advancing CZP toward rational clinical evaluation.

## 5. Human-Relevant Translational Strategies to Advance Clozapine Repurposing

The limitations outlined in the previous section collectively point toward a common requirement: experimental and computational frameworks that more faithfully capture human tumor biology, pharmacokinetic complexity, and interindividual variability [97,98]. To our knowledge, no such framework has yet been developed for CZP in an oncological context; however, this gap highlights a clear translational opportunity for future research. Patient-derived tumor organoids, advanced co-culture systems, and microphysiological platforms may serve as complementary tools to improve translational relevance. By preserving key aspects of human tumor architecture, cellular heterogeneity, and stress-response capacity, these systems enable evaluation of drug effects under conditions that more closely approximate human biology [99]. In the context of CZP, they offer a particularly useful opportunity to assess antitumor activity and toxicity-related endpoints within the same human-derived system [78,99,100], which is especially relevant for a compound in which biological efficacy and adverse responses are closely linked. These platforms may also provide a practical means to evaluate the combination and targeted delivery strategies discussed in Section 4 at pharmacologically achievable concentrations, enabling identification of tumor contexts with enhanced sensitivity and assessment of synergistic regimens designed to potentiate anticancer effects at sub-therapeutic CZP doses, thereby reducing the risk of systemic toxicity. By generating multilevel outputs, including functional ex vivo readouts and omics data, these platforms may also help clarify the mechanisms of response and identify clinically actionable vulnerabilities [82,83]. It should be acknowledged that these platforms have limitations, including a lack of a fully tumor microenvironment, particularly functional immune and vascular components, while microphysiological platforms remain technically demanding and difficult to standardize across laboratories. These constraints do not undermine their translational value but underscore the importance of interpreting their outputs within a broader experimental framework. Even so, the biological signals generated by these systems may provide a grounded basis for subsequent mechanistic modeling and patient stratification.

While human-relevant experimental systems address the biological gap between conventional models and human tumor biology, they do not fully resolve the challenge of predicting individual patient response to drug exposure or optimizing dosing strategies prior to clinical testing [101,102]. In this context, computational modeling frameworks—and digital twin (DT) approaches in particular—may serve as complementary tools to extend experimental findings into the in silico domain. DTs typically rely on the integration of multimodal data, including genomic profiles, signaling network architecture, imaging features, and clinical parameters, to generate individualized representations of tumor behavior and treatment response under different therapeutic scenarios [101,102]. Although virtual twin approaches have been explored for CZP in psychiatric settings [103,104], their application to oncology remains speculative at this stage. Nevertheless, these prior studies provide a technical precedent for patient-specific CZP modeling and suggest that a future CZP-focused DT framework could, in principle, be adapted to oncological contexts. If developed for CZP in oncology, such a framework would need to be grounded in the tumor contexts and molecular factors already identified throughout this review. Potential inputs could include organoid-derived readouts of viability, apoptosis, autophagy, and mitochondrial integrity from the GBM, MBM, NSCLC, and breast cancer models discussed in Section 3; transcriptomic and proteomic data capturing context-dependent activation of PI3K/Akt signaling, autophagic flux, inflammation-related pathways, and angiogenic programs; and patient-level toxicological parameters such as CYP1A2 metabolic capacity, inflammatory status, and baseline hematological risk. These data could then be incorporated into mechanistic or knowledge-based network structures to support tumor-context stratification by vulnerability profile, simulation of exposure–response relationships at clinically achievable concentrations, and early estimation of hematological and metabolic toxicity risk. In this sense, a CZP-related DT would be expected to inform several translational questions, including therapeutic efficacy, toxicity, patient stratification, and treatment optimization, rather than serving as a replacement for empirical validation [105,106,107,108].

More broadly, the value of this approach lies in its potential to explore efficacy and toxicity jointly through virtual assessment of dosing regimens, toxicity thresholds, and response scenarios, thereby informing translational planning while limiting unnecessary early-stage clinical exposure. Collectively, human-relevant systems and DT frameworks should be viewed as complementary rather than independent strategies. The former can generate biologically grounded outputs under controlled conditions, while the latter can integrate those outputs into patient-specific predictive models that support hypothesis generation, dose reasoning, and prioritization of future translational studies. In this sense, the combined use of both approaches may be useful to define which CZP responses warrant progression toward more advanced preclinical validation, and eventually, clinical investigation [108,109,110]. In this sense, the combined use of both approaches may help define which CZP responses warrant progression toward more advanced preclinical validation and, ultimately, clinical investigation.

**Figure 3:**
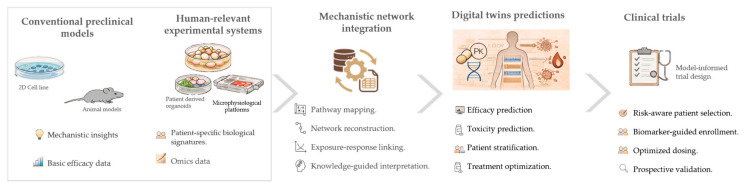
Proposed translational roadmap from conventional preclinical models to DT-informed clinical trial design. Each stage builds upon the outputs of the preceding one. Conventional preclinical models provide initial mechanistic insights and basic efficacy signals, while human-relevant experimental systems, including patient-derived organoids, co-cultures, and microphysiological platforms, generate multimodal experimental outputs that capture tumor-context dependency and exposure-related phenotypes. These data can then be integrated through mechanistic, network-based structures to support a prospective CZP DT capable of simulating exposure–response relationships, predicting efficacy and toxicity, and informing tumor-context stratification and treatment optimization. In the final stage, such a framework could support risk-aware patient selection, biomarker-guided enrollment, and prospective validation in future clinical studies. Together, these complementary components may help bridge the gap between experimental findings and clinically actionable evidence, thereby informing rational and risk-aware clinical trial design. The figure should be interpreted as a conceptual and forward-looking translational roadmap rather than as evidence that an oncology-specific clozapine digital twin is already established.

The absence of studies combining human-relevant models and DT frameworks for CZP in an oncological context highlights the opportunity for a future roadmap in which these approaches are used as complementary tools to advance CZP research and, ultimately, its translational potential. Figure 3 summarizes this proposed roadmap, illustrating how human-relevant experimental systems and DT frameworks may be integrated to support future CZP studies in oncology.

## 6. Conclusions

Clozapine illustrates both the promise and the complexity of repurposing pharmacologically pleiotropic agents for oncology. Its broad receptor engagement, downstream signaling effects, and capacity to disrupt cellular stress tolerance have generated anticancer signals across several experimental systems. However, these effects cannot be interpreted independently of its behavior as a xenobiotic, including its complex biotransformation, exposure-dependent pharmacology, metabolite formation, systemic adverse-effect profile, and clinically relevant hematological liabilities. Rather than acting as a conventional cytotoxic agent with a single dominant mechanism, CZP may be more appropriately viewed as a context-dependent modulator of proliferative equilibrium, metabolic resilience, and stress adaptation.

The principal translational challenge is therefore not limited to demonstrating anticancer activity. It is necessary to determine whether the exposures required to affect tumor cells can be achieved without producing unacceptable toxicity, while accounting for pharmacokinetic variability, metabolic differences, drug–drug interactions, and individual susceptibility to adverse reactions. This requires a clear distinction between potentially useful tumor-selective pharmacology and nonspecific xenobiotic-induced cellular stress. It also requires the prospective identification of tumor contexts in which CZP-related vulnerabilities are most likely to translate into a meaningful therapeutic window. Such contexts are unlikely to be defined by a single biomarker, but rather by composite molecular, metabolic, pharmacological, and toxicological signatures.

The integration of patient-derived experimental models, microphysiological systems, pharmacokinetic–pharmacodynamic and toxicokinetic modeling, and digital twin frameworks offers a rational strategy to address these questions. These approaches may enable the stratified evaluation of dose–exposure relationships, metabolic liabilities, combination regimens, tumor response, and patient-specific toxicity risk within biologically relevant settings. Importantly, these tools do not replace experimental or clinical validation; instead, they can refine their direction by identifying the most informative models, exposure ranges, biomarkers, and patient subgroups for further investigation.

More broadly, CZP highlights an emerging principle in drug repurposing: the transition from an established therapeutic drug to a potential anticancer agent requires an integrated understanding of both pharmacological activity and xenobiotic behavior. For centrally acting agents with complex systemic effects, successful translation will depend on frameworks that bridge drug metabolism, tumor biology, exposure–response relationships, toxicology, and patient-specific variability. Whether CZP will ultimately fulfill its preclinical promise in oncology remains uncertain. Nevertheless, the evidence reviewed here supports its value as a scientifically informative case study and justifies further investigation through a biologically informed, model-guided, and risk-aware translational strategy.

## Figures and Tables

**Table 1:** Summary of preclinical evidence for CZP anticancer activity across tumor models.

Cancer Type/Model	Experimental System	Drug Concentration	Main Biological Effects	Proposed Mechanism of Action	Ref.
Glioblastoma	U-87MG (PTEN-deficient human GBM cells)	20–40 µM	Inhibition of proliferation; G_0_/G_1_ cell-cycle arrest	Suppression of PI3K/Akt signaling and downstream cell-cycle control	[48]
Glioblastoma/ Neuroblastoma	A172 (glioblastoma), SH-SY5Y (neuroblastoma)	0–100 µM	Reduced cell viability and metabolic activity; maintained cytotoxicity under oxidative stress	Not clearly defined in the study	[49]
Melanoma brain metastases	Human MBM cell lines; human astrocytes; rat brain organoids	~10–40 µM (IC_50_ ≈ 26–29 µM)	↓ proliferation, migration and clonogenicity; ↑ apoptosis; tumor selectivity vs. non-malignant cells	Modulation of survival, inflammatory, and angiogenic pathways	[50]
Melanoma metastatic	M1/15 human metastatic melanoma xenografts (athymic nude mice)	1 mg/kg (s.c.)	↓ tumor growth; ↑ median survival; ↓ mitotic index and angiogenesis	Histamine receptor-associated antitumor effects	[51]
Non-small cell lung cancer	A549, H1299	0–50 µM	↓ proliferation; G_0_/G_1_ arrest; extensive vacuolization; autophagy	Autophagy-associated growth suppression	[52]
Breast cancer (ER+)	MCF-7	0–50 µM	↓ proliferation and clonogenic survival; G_0_/G_1_ arrest; autophagy and apoptosis	ROS-driven oxidative stress; autophagy as survival response	[53]
Breast cancer (TNBC)	MDA-MB-231	0–100 µM	↓ viability; cell-cycle arrest; mitochondrial dysfunction; apoptosis	Mitochondria-dependent intrinsic apoptotic signaling	[54]

ER+, estrogen receptor-positive; GBM, glioblastoma; IC_50_, half-maximal inhibitory concentration; PTEN, phosphatase and tensin homolog; s.c., subcutaneous; TNBC, triple-negative breast cancer; ↓ decrease; ↑ increase.

**Table 2:** Summary of current translational constraints limiting the clinical development of CZP as an anticancer agent.

Domain	Constraint	Translational Implication	Ref.
Safety and tolerability	Risk of agranulocytosis and severe neutropenia requiring mandatory blood monitoring	Limits use in myelosuppressed oncological populations; requires continuous vigilance	[69,70]
	Associated adverse effects include myocarditis, cardiomyopathy, and metabolic disturbances		[71,72,73]
Pharmacokinetic variability	Substantial interindividual variability driven by CYP1A2 metabolism, influenced by smoking status, inflammation, and co-medication	Complicates dose optimization; requires therapeutic drug monitoring	[74,75]
Concentration gap	Anticancer-effective concentrations in vitro (10–100 µM) substantially exceed therapeutically achievable plasma levels (0.8–1.8 µM)	Raises fundamental questions regarding clinical translatability of in vitro findings	[73,76,77]
Dose-toxicity	Achieving anticancer-relevant concentrations would require doses exceeding psychiatric practice, amplifying toxicity risk	Defines a narrow and currently undefined therapeutic window	[71,72,73]
Preclinical models	Conventional models fail to capture human-specific efficacy–toxicity relationships and interindividual variability	Constrains translational value of existing preclinical data	[78,79,80,81]
PK/PD modeling	Absence of integrated PK/PD models linking plasma exposure to antitumor effect	Exposure–response relationship remains unquantified	[82]
Clinical evidence	No clinical trials conducted; available human data limited to retrospective observational studies	Insufficient to inform therapeutic conclusions; efficacy in humans undemonstrated	[83]

## Data Availability

No new data were created or analyzed in this study. Data sharing is not applicable to this article.

## Abbreviations

The following abbreviations are used in this manuscript:

CZP	Clozapine
DT	Digital Twins
MBM	Melanoma brain metastasis
PTEN	Phosphatase and TENsin homolog
EGF	Epidermal growth factor
NHA	Non-malignant human astrocytes
MIP-1α	macrophage inflammatory protein-1α
IL-8	interleukin-8
ANGPTL-4	angiopoietin-like 4
NSCLC	non-small cell lung cancer
PK/PD	pharmacokinetics/pharmacodynamics
PCNA	proliferating cell nuclear antigen
